# Current Possible Newer Pharmacological Treatment Options for Alzheimer’s Disease

**DOI:** 10.3390/ijms27219959

**Published:** 2026-11-06

**Authors:** Barbara Miziak, Ryszard Pluta, Stanisław J. Czuczwar

**Affiliations:** Department of Pathophysiology, Medical University of Lublin, 20-090 Lublin, Poland; pluta2018@wp.pl

**Keywords:** Alzheimer’s disease, anti-tau protein monoclonal antibodies, bezisterim, posiphen, ACD856, tenuigenin, gemfibrozil, coumarin derivatives, ponicidin, salicin

## Abstract

Alzheimer’s disease (AD), as a chronic neurodegenerative disorder, leads to progressive dementia. About 60–80% of dementia cases are due to AD, and their incidence increases especially after the age of 65. AD is characterized by impaired memory, abnormal behavior, and impaired thinking process, which causes limitation of daily activities and a gradual loss of independence. The costs of all dementia cases are likely to reach $2 trillion in 2030 alone. Currently available pharmacological treatment of AD is, for the most part, not causal, and does not modify the course of the disease. The data search for procognitive drugs was carried out using the PUBMED and Clinical Science databases. Among the drugs with animal data and preliminary clinical trials, the following can be distinguished: fosgonimeton, blarcamesine, bezisterim, posiphen (buntanetap) and ACD856. As a rule, promising preclinical results were not confirmed in clinical trials. Some clinical expectations may be associated with posiphen and ACD856. Based on preclinical data, the following drugs seem promising: tenuigenin, JQ1, gemfibrozil, coumarin derivatives, ponicidin, salicin, calcaratarin D, new 1,3,5-triazines or 2-aminopyrymidine carboxamide derivatives, epimedium brevicornu flavonoids, and drugs affecting the glymphatic system. In general, the above-mentioned drugs counteract the pathologies leading to the occurrence of AD.

## 1. Introduction

Alzheimer’s disease (AD) is a chronic neurodegenerative disorder, leading eventually to dementia. The neurodegenerative process starts in the hippocampal area, the CA1 field being the most affected [1]. Approximately, 60–80% of dementia cases may be attributed to AD, the prevalence of which is significantly higher in patients aged 65 and older. In the group of patients aged 85 and older, approximately 50% of cases of AD are reported. Currently, the number of AD patients reaches 50 million worldwide, and, by 2050, it is expected to reach 140 million [2]. The costs of different types of dementia worldwide are enormous, and amounted to $ one trillion in 2018 alone, likely reaching two trillion US dollars in 2030 [3]. AD begins with impairments in memory, behavior, and thinking, gradually leading to a reduction in patients’ daily activities and loss of independence [2]. Finding drugs that would significantly slow the progression of AD is therefore an urgent priority.

## 2. Search Methods

Potential drugs that could improve the condition of AD patients (in terms of memory and cognition) were searched in English language articles in the following computer databases: PUBMED (for clinical and experimental data) and Clinical trials (for clinical data). The following keywords were used: AD and novel (innovative) treatment, AD and clinical trials. Particularly relevant references were also extracted from publications found in the above databases. This is a narrative review, and no predefined inclusion/exclusion criteria were used. When searching the databases, the focus was on the most recent research publications, meta-analyses and review articles, mainly from the period 2021–2026.

## 3. Suggested Causes of AD

There are several hypotheses related to the pathogenesis of AD, which may indicate the involvement of multiple factors in the development of this disease. The cholinergic hypothesis is based on the observation of increased activity of acetylcholinesterase and butyrylcholinesterase (enzymes that break down acetylcholine), which leads to decreased activity of the cholinergic system in the brain cortex. It is this cortical deficit in cholinergic neurotransmission that, according to this hypothesis, leads to dementia and typical behavioral disturbances in AD [4].

Another hypothesis assumes that the factor responsible for the occurrence of AD is β-amyloid (Aβ), which is produced by the action of two enzymes, β-secretase (BACE1) and γ-secretase, on the amyloid precursor protein (APP). By deposition in the brain in the form of insoluble fibrils, this causes neuronal loss. Aggregated and deposited Aβ (senile plagues) is also involved in the process of neuroinflammation and oxidative stress, which causes further progression of AD [5]. Interestingly, microglia-dependent neuroinflammation can be considered a pathogenetic factor in AD that, through the release of pro-inflammatory cytokines, may exacerbate the deleterious effects of Aβ and tau protein [6]. In addition, it has been shown that acetylcholinesterase not only increases Aβ aggregation, but also exacerbates its neurotoxicity [7].

The third hypothesis points to tau protein, whose hyperphosphorylated form is no more capable of stabilizing cytoskeletal microtubules and, moreover, is responsible for the formation of neurofibrillary tangles [8]. Ultimately, hyperphosphorylated tau protein, through a number of kinases (for instance, glycogen synthase kinase-3β), causes profound neuronal degeneration [9,10]. Remarkably, there is evidence that Aβ deposits precede the formation of neurofibrillary tangles [11].

Oxidative stress plays a significant role in many neurological diseases, and it seems that, also in AD, reactive oxygen species lead to neurodegenerative processes by damaging cellular proteins, lipids and DNA [12]. Reactive oxygen species have also been found to stimulate enzymes synthesizing Aβ and formation of senile plaques [8].

Pluta et al. [13,14] demonstrated that rats that survived an episode of cerebral ischemia-reperfusion, and which were followed for up to 2 years post-ischemia, developed brain atrophy and dementia. Interestingly, ischemia-induced brain neurodegeneration showed striking similarities to that caused by AD. Furthermore, Aβ and tau-protein deposits were found in the brains of rats after ischemia. All of the above changes were accompanied by dysregulation of genes closely associated with AD, including the genes for APP, α-secretase, β-secretase (BACE1), presenilin 1 (PSEN1) and preselin 2 (PSEN2) and tau protein. The latest research in this field reveals the influence of ischemia on a number of other AD-associated genes—e.g., for apolipoproteins (APOA1, APOE, CLU), α-synuclein, and mitophagy (BNIP3), apoptosis (CASP3) and autophagy (BECN1), in the brain [15,16,17,18].

## 4. Currently Used Drugs to Treat AD

According to the cholinergic hypothesis, cholinesterase antagonists, including donepezil, galantamine, and rivastigmine, can be used to treat AD [19]. All three drugs significantly improved cognitive performance in patients with AD. Galantamine was well-tolerated, whereas gastrointestinal side effects were observed with rivastigmine. Donepezil is contraindicated in patients with swallowing disorders [19]. These drugs may be prescribed for all stages of AD, but are most effective in the early stages. Unfortunately, they do not inhibit the progression of AD [20].

Memantine, as an N-methyl-D-aspartate receptor antagonist, ameliorates some cognitive aspects of AD patients [20] in moderate and severe cases of AD. The drug may also be combined with donepezil [21]. According to the Severe Impairment Battery score, this combination resulted in a better therapeutic effect when compared to either drug alone. However, this beneficial result did not occur when measured by Mini-Mental State Examination (MMSE) score. The most frequent adverse effects for the combined treatment were dizziness and hearth rhythm disturbances [21].

Recently, anti-amyloid monoclonal antibodies such as aducanumab and lecanemab have been approved in the USA for the management of mild stages of AD [22,23]. Aducanumab was effective in reducing Aβ plaques. Unlike the symptomatic medicines mentioned above, this drug was, for the first time, able to slow down the progression of AD. However, due to some problems in phase 3 clinical trials, aducanumab, as a not entirely convincing medicine, has not been approved in Europe. As for lecanemab, in a phase 3 clinical trial lasting for 18 months, it showed a significant slowing of dementia compared to the placebo group. In addition, clinical and biomarker secondary endpoints were also demonstrated to be met with this drug. Unlike aducanumab, lecanemab has also been approved in Europe [23]. However, there is still debate as to whether the benefits outweigh the risks of using anti-amyloid therapy [22].

## 5. Potential Drugs for the Treatment of AD with Available Clinical Data

### 5.1. Anti-TauProtein Monoclonal Antibodies

As already mentioned, the tau protein is involved in stabilizing cytoskeletal microtubules, which supports cellular structure and intracellular transport [8]. Additionally, hyperphosphorylation of tau protein is responsible for its reduced affinity to microtubules, which results in increased concentration of intracellular tau protein, causing damage to euronal function, and, consequently, neurotransmission [24]. It therefore appears that blocking tau-protein hyperphosphorylation may inhibit the above-mentioned consequences.

The network meta-analysis study analyzed 2193 patients from six studies designed to test the efficacy and safety of an anti-tau protein monoclonal antibody for the treatment of AD [25]. Of the four drugs evaluated (gosuranemab, semorinemab, tilavonemab, and zagotenemab), semorinemab had the best profile compared to other anti-tau protein drugs, in terms of improving MMSE scores and reducing the score on the Alzheimer’s Disease Assessment Scale—Cognitive (ADAS-Cog). Zagotenemab also outperformed the placebo on the MMSE. Regarding the ADAS-Cog scale, the best results were achieved for semorinemab, tilavonemab, and zagotenemab. Surprisingly, the placebo group had the best result on the Clinical Dementia Rating Scale Sum of Boxes (CDR-SB), followed by tilavonemab and zagotenemab. As regards adverse or serious adverse effects, there was no significant difference in their induction by anti-tau protein drugs. Among these, tilavonemab had the lowest impact on adverse events and serious adverse events, falls, and urinary tract infections, compared to the placebo group [25,26,27,28,29,30]. In addition to the assessment of broadly defined adverse effects, the following were also assessed: amyloid-related imaging abnormalities with edema or effusions (ARIA-E) and amyloid-related imaging abnormalities with hemosiderin deposits (ARIA-H). Tilavonemab had the lowest incidence of ARIA-E and ARIA-H among anti-tau protein drugs. However, it should be emphasized that such side effects were observed in only 0.3% of patients.

### 5.2. Ligands of 5-HT6 Receptors

There are multiple types of serotonin (5-HT) receptors, and the 5-HT6 receptor is coupled to the G-protein/adenylyl cyclase3 isoform pathway. This receptor has been found in many brain regions, including the cortex, hippocampus, thalamus, striatum, and cerebellum. Interestingly, its peripheral expression is low, so the occurrence of possible serious peripheral adverse effects of potential drugs acting on 5-HT6 receptors is unlikely [31]. Preclinical studies indicate, strikingly, that both agonists and antagonists of this receptor elicit similar behavioral responses [32]. Namely, they were documented to alleviate scopolamine- or MK-801 (an N-methyl-D-aspartate antagonist)-induced memory impairment in rats, which may be ascribed to their enhancement of acetylcholine- and glutamate-mediated events. Probably, a similar effect may be the result of interaction with 5-HT6 receptors located on glutamatergic and cholinergic neurons or GABAergic interneurons involved in indirect disinhibition of glutamatergic/cholinergic neurons [33,34].

The clinical evidence for most 5-HT6 receptor antagonists (latrepirdine, intepirdine, and idalopirdine) is rather disappointing. Only masupirdine performed better than placebo, in a number of indicators—ADAs-Cog, CDR-SB or MMSE [35].

### 5.3. Fosgonimeton

Fosgonimeton was developed to enhance the action of hepatocyte growth factor and its associated receptor—the mesenchymal–epithelial transition factor. Its centrally-mediated effects resulted in clear-cut neuroprotection in a number of neurotoxicity models. Interestingly, the drug also elevated mitochondrial membrane potential and improved mitochondrial functions [36]. It is noteworthy that drug-induced stimulation of the neurotrophic system may enhance cognition, probably via restoring synaptic connections [37,38]. In preclinical models of dementia, this drug was shown to have pro-cognitive and neurotrophic effects. The mechanism responsible worked through counteracting the toxic effects of Aβ [39].

The drug was investigated in phase 1 and phase 2 clinical trials [40,41]. It was found to be well-tolerated across the entire dose range, and its pharmacokinetics was dose-proportional. Importantly, normalization of the assessed EEG parameters was also observed in AD patients. In particular, the normalization of the event-related potential P300 (ERP P300) latency in the EEG supports the procognitive effect and enhancement of synaptic function by fosgonimeton [40]. In a phase 2 clinical trial, this drug was used in patients with mild-to-moderate AD [41]. At baseline, ERP P300 latency showed no significant differences across the study group. After 26 weeks of taking the drug, improvement was observed in ERP P300 latency and on the ADAS-Cog11 scale. No serious adverse events were observed. The most common adverse event was a skin reaction at the injection site. Clinical trials are continuing [41].

### 5.4. Blarcamesine—A Sigma-1 Receptor Agonist

Sigma-1 receptors are located in the endoplasmic reticulum, and regulate mitochondrial activity, proteostasis, and calcium homeostasis. Moreover, their stimulation is associated with the intensification of autophagy. Further, autophagy is responsible for clearing misfolded proteins like, for instance, tau-protein aggregates [42].

Blarcamesine was administered to mice daily for 7 days before intracerebroventricular administration of the amyloidogenic Aβ fragment. The studies were performed 7 days after toxin administration. Memory tests showed significant attenuation of the adverse effects of the amyloidogenic Aβ fragment by blarcamesine. Furthermore, blarcamesine significantly inhibited the increase in lipid peroxidation in the hippocampus [43].

In a randomized, placebo-controlled, clinical-phase-2B/3 trial, patients with early stages of AD were given oral blarcamesine for 48 weeks. ADAS-Cog13 and CDR-SB scales showed a significant improvement at *p* < 0.025. Only the Alzheimer’s Disease Cooperative Study—Activities of Daily Living scale (ADCS-ADL) outcome did not differ significantly. As regards whole-brain volume loss after 48 weeks, blarcamesine very significantly inhibited this parameter, at *p* < 0.002. Moreover, plasma Aβ42/40-ratio was significantly elevated by the drug (*p* = 0.048). Treatment-related serious adverse effects (≥1) were recorded in 56 patients (16.7%), compared to 17 (10.1%) in the placebo group. The results clearly indicate that blarcamesine at 30 and 50 mg significantly slowed progression of AD, by 34.6 and 38.5%, respectively, versus the placebo group [44].

### 5.5. Bezisterim

Bezisterim (NE3107) is closely related to the adrenal sterol metabolite 5-androstene-3beta,7beta,17beta-triol [45]. It is an inhibitor of toll-like receptor 4 (TLR4)—and tumor necrosis factor-induced activation of NF-κB and a reducer of extracellular signal-regulated kinase (ERK) phosphorylation, eventually leading to a reduction in the expression of proinflammatory chemokines and cytokines [46]. In an animal model of Parkinson’s disease, the drug exhibited neuroprotective activity [47]. In a phase 2, open-label study, bezisterim was administered for 3 months to 23 patients with mild cognitive impairment or mild dementia [48]. In 17 patients with MMSE ≥ 20, statistically significant improvements were noted in ADAS-Cog 11, Clinical Dementia Rating, Quick Dementia Rating Scale, Alzheimer’s Disease Composite Score, and clinician-rated GRC. Also, there was a beneficial modification of fluid P-tau protein levels and P-tau protein/amyloid beta 1–42 ratio. The drug was also evaluated in a trial involving 439 patients with mild-to-moderate probable AD, who were randomly assigned to two groups (1:1) receiving either bezisterim or placebo [49]. In 57 patients from the bezisterim group vs. 50 from the placebo group, metabolic and inflammatory parameters were assessed, along with analysis of plasma biomarkers. It turned out that bezisterim administration resulted in statistically insignificant improvements in cognitive and functional performance, as demonstrated by primary and secondary endpoints. These improvements were correlated to biomarkers of metabolism, dementia, and inflammation [49].

### 5.6. Posiphen (Buntanetab)

Posiphen{(+)-phenserine} is actually the positive enantiomer of (-)-phenserine, which is an inhibitor of acetyl-cholinesterase. However, posiphen does not have this particular feature, but effectively reduces the levels of APP and Aβ42 in cultured human neuroblastoma cells by apparently decreasing the APP synthesis [50]. The possibility of obtaining this effect in vivo was investigated in mice given posiphen at doses of 7.5–75 mg/kg daily, for 21 days. Indeed, the results indicated that posiphen did decrease the brain concentrations of total APP in a dose-dependent manner and, in the dose range of 15–75 mg/kg, it also lowered the brain levels of Aβ40 and Aβ42. Among the brain secretases, only the activity of β-secretase was reduced [50]. Posiphen was also tested in the APP/PS1 mouse model of AD [51]. These transgenic mice are characterized by high concentrations of Aβ42, the early appearance of senile plaques, and cognitive impairment [52]. Oral administration of the drug was initiated in transgenic two-month-old mice, behavioral and electrophysiological tests being performed in three-month-old animals [51]. Administration of posiphen resulted in the restoration of spatial memory and improvement in contextual spatial learning. Moreover, treatment with posiphen restored normal long-term potentiation in hippocampal slices of the transgenic mice, which could be interpreted as an improvement in synaptic function. For the estimation of brain APP and all related peptides, transgenic mice were given posiphen for three weeks, at 75 mg/kg (for the first week) and 50 mg/kg for the rest of the time. It turned out that posiphen significantly reduced the hippocampal level of APP by 40% and that of Aβ42 by 68% [51].

Clinical data are also available. Posiphen or placebo for 25 ± 2 days were evaluated in 14 early-AD patients [53]. The patients’ cognition was significantly improved, as measured in ADAS-Cog11 and Wechsler Adult Intelligence Scale (WAIS) coding tests. Biomarker analysis led to the conclusion that the drug treatment led to improving axonal integrity and synaptic function in AD patients [53]. In a double-blind phase 1b randomized clinical trial, posiphen 60 mg was given once daily (5 patients) or twice daily (5 patients), and a placebo (5 patients), for 21–23 days, to patients with mild cognitive impairment or mild AD [54]. Dose-dependent lowering of APP synthesis, as revealed in cerebrospinal fluid (CSF) samples, was observed. However, posiphen did not affect the fractional synthesis rate for CSF Aβ40 vs. placebo group. Also, posiphen did not significantly influence cognitive measures, as compared with the placebo [54]. Posiphen (in a phase 2/3 study) was also administered to 318 patients with early-stage AD at an oral daily dose of 15 mg (33 patients were homozygous for *APOE4*, 126 were heterozygous, and 159 were non-carriers). The positive results observed on the ADAS-Cog 11 scale, compared to the placebo group, were independent of *APOE4* status after 12 weeks and 3 months [55].

### 5.7. Positive Allosteric Modulator of Neurotrophin Receptors

Recently, a triazinetrione derivative, ACD856, a positive allosteric modulator of pan- tyrosine kinases (Trk) receptors, was evaluated for its neuroprotective activity and likely disease-modifying potential [56,57]. Signaling via neurotrophin receptors was clearly enhanced by ACD856, with its EC50 values of 0.38 and 0.30 µM for TrkA or TrkB receptors, respectively [56]. It is noteworthy that single administration of the modulator resulted in an enhancement of cognitive function in 18-month-old mice, which could function as well as 2-month-old mice [56]. Its effects were evaluated in recombinant cell lines, primary cerebral cortex neurons, and in vivo [57]. ACD856 was found to enhance nerve growth factor -induced neurite outgrowth. In PC12 cells, it increased the concentration of the presynaptic protein SNAP25, and in SH-SY5Y cells it increased the level of phosphorylated TrkB. In primary cortical cells, ACD856 increased the levels of phospho-ERK1/2 and brain-derived neurotrophic factor (BDNF), demonstrating neuroprotective effects against Aβ (or energy deprivation)-induced neurotoxic effect. A significant increase in BDNF levels was also observed in the brains of aged (21-month-old) mice. Results of subsequent experiments demonstrated antidepressant effects, improved memory, and increased neuroplasticity, after administration of ACD856 [57].

ACD856 was initially tried in six healthy volunteers (*N* = 6) in a microdose of 0.1 mg. Then, a randomized phase 1, double-blind, placebo-controlled trial (*N* = 56) followed, with the modulator at a single dose of 1 to 150 mg [58]. The drug was well-tolerated, with no adverse effects within this dose range. The pharmacokinetic parameters were bi-exponential plasma decline, low distribution volume and plasma clearance, and a half-life reaching 20 h. After oral administration, the drug was rapidly absorbed, and almost complete bioavailability was observed. Food intake had no significant effect on plasma half-life of ACD856 or on its bioavailability [58].

### 5.8. Inhibitors of Programmed Cell Death Protein (PD-1) and Its Ligand (PD-L1)

PD-1/PD-L1 signaling plays a role in tumor immunity. PD-1/PD-L1 interaction blockade abolishes cancer immune evasion, restoring the anti-tumor immune response [59]. Yoon et al. [60] have reviewed available data on the influence of the inhibitors of the PD-1/PD-L1 pathway on AD-associated pathology and cognition in animal models of AD. Preclinical study results clearly indicate that PD-1/PD-L1 inhibitors led to a reduction in Aβ plaque burden, tau-protein phosphorylation, and astrocyte reactivity. Moreover, reduction in AD pathology was accompanied by improvement in cognitive performance in animal models of AD. Interestingly, the tested inhibitors did not affect cognitive processes in naïve animals.

Although the effect of PD-1/PD-L1 inhibitors on dementia patients has not been studied, there are results of studies on cognitive processes in cancer patients. Results of a meta-analysis based on 40 studies indicate that these inhibitors had a beneficial effect on cognitive function in cancer patients [60].

### 5.9. Tenuigenin and β-Asarone

Tenuigenin may play a significant role in both treatment and prevention, and even exhibits neuroprotective potential in AD. The drug may inhibit the aggregation of Aβ and tau proteins; it exhibits anti-inflammatory and antioxidant effects, counteracts neuronal apoptosis (by, among other things, increasing levels of superoxide dismutase, glutathione, and mitochondrial membrane potential), and enhances the functioning of the cholinergic system [61].

Mitogen-activated protein kinase 1 (MAP2K1) has been identified as a critical target for tenuigenin. Moreover, gene-set enrichment analysis provided evidence that tenuigenin, by activating 26S proteasome, may lead to degradation of neurotoxic proteins, including Aβ [62]. Immune infiltration analysis showed that tenuigenin, by modulating the distribution of M0 macrophages and natural killer cells, restored immune balance in the AD microenvironment. Strong binding affinity of this agent to MAP2K1 was confirmed by molecular docking. Eventually, in vitro studies revealed that in Aβ-treated SH-SY5Y cells, tenuigenin distinctly inhibited the expression of MAP2K1 protein and increased cell viability [62].

In another study, using a rat model of AD (induced by the administration of streptozotocin into the brain ventricles), tenuigenin was administered for 4 weeks. Improvements were observed in behavioral tests, including cognitive function, a reduction in selected markers of oxidative stress, and the inhibition of changes associated with tau hyperphosphorylation [63].

β-Asarone, a plant-derived agent, was administered intragastrically to APP/PS1 transgenic mice, and then the animals were evaluated in water maze, passive avoidance, and step-down tests for spatial learning and memory [64]. It turned out that β-asarone significantly improved the performance of transgenic mice in the above tests, when compared to the respective controls. After all, one month’s treatment with this agent decreased hippocampal acetylcholinesterase and Aβ42 concentrations, and elevated the expression of phosphorylated mechanistic target of rapamycin (m-TOR) and p62, reducing that of phosphorylated Akt, beclin-1, and LC3B proteins. Also, hippocampal APP mRNA and beclin-1 mRNA were reduced by the treatment. The authors are of opinion that the procognitive effect of β-asarone may result from reducing beclin-1 dependent autophagy through the P13K/Akt/m-TOR pathway [64].

It appears that β-asarone may also have other mechanisms of action. Yang et al. [65] confirmed its beneficial cognitive effects in 3xTg-AD mice. Evidence was presented that this agent reduced hippocampal neurodegeneration and reduced both Aβ deposition and tau-protein hyperphosphorylation in hippocampal CA3 neurons of 3xTg-AD mice. In vitro studies in BV-2 cells showed that β-asarone upregulated the triggering receptor expressed on myeloid cells 2 (TREM2), and caused activation of the P13K/Akt pathway and inhibition of GSK3β activity. These particular actions led to the change of microglia pro-inflammatory M1 phenotype to the anti-inflammatory M2 phenotype, thus reducing the neuroinflammatory process [65]. Xu et al. [66] also tested β-asarone in 3xTg-AD mice and BV-2 cells. In vivo, this agent was administered for 8 weeks, and then the animals were evaluated in the Morris water maze and step-down test. Learning and memory were considerably increased in treated mice. In the hippocampus of 3xTg-AD mice, the treatment resulted in less neuronal loss. Also, the expression of Aβ, APP, and tau-protein phosphorylation was significantly reduced. Both, in the hippocampus and BV-2 cells β-asarone, downregulated neuroinflammatory markers and modulated proteins associated with autophagy (beclin-1, P62, ATGS, LC3-II/I). In addition, components of the nucleotide-binding domain and leucine-rich repeat protein 3 (NLRP3) inflammasome were distinctly suppressed [66].

Both of these agents were also added to memantine-treated AD patients [67,68]. In the first study [67], 48 patients were administered β-asarone and tenuigenin, as well as memantine, at a daily dose of 5–20 mg. The control group consisted of 45 patients and received memantine only at the above dose. The average scores in both groups, before adding β-asarone and tenuigenin, were similar. The additional treatment lasted for 12 weeks. Mini-Mental State Examination (MMSE), Activities of Daily Living (ADL) and Clinical Dementia Rating Scale (CDR) scores improved very significantly in the experimental group, without any enhancement of drug-dependent adverse effects. In the second study, the results were quite similar, with significant improvements in the above scores and comparable adverse effects. The authors of this study accentuate the fact that the added agents improved the therapeutic effects of memantine in moderate-to-severe AD cases [68].

Based on the available data, it can be concluded that tenuigenin is an interesting pre-clinical candidate with potential multi-faceted effects in the context of AD, which justifies further research. It should be borne in mind, however, that at this stage, tenuigenin cannot be regarded as a substitute for standard treatment, nor can it be treated as a proven method of preventing AD.

The situation is similar in the case of β-asarone; the aforementioned studies also point to its multifaceted nature in relation to AD. It is worth noting, however, that both substances are of plant origin, which may be associated with a potential risk of toxicity. There are, however, few studies on this subject, and the available data often do not provide clear-cut results, and originate from older studies. Uebel et al. [69] analyzed, amongst other things, studies on the mutagenicity, genotoxicity, chronic toxicity, organ toxicity, effects on reproduction and carcinogenic potential of three asarone isomers, including studies on β-asarone. Studies, mainly in rodents, indicate a high likelihood of carcinogenic effects in rodents (at 52 and 104 mg/kg, i.p.). This hypothesis has been confirmed in vitro, where it has been shown that the propenyl side chain of β-asarone tends to be activated to form epoxides, which, in turn, are capable of reacting with DNA, resulting in genotoxic and, consequently, mutagenic effects [69]. In contrast, other studies [70] have shown that in mice treated with β-azarone (182.4 mg/kg, i.p.), no genotoxicity or changes in the polychromatic erythrocyte pattern were observed in femoral bone-marrow samples.

Studies of the subacute toxicity of β-asarone (100 mg/kg, i.p., for 5 consecutive days) in Sprague-Dawley rats (prior to weaning) pointed to a decrease in body weight, associated with reduced food intake (with no effect on mortality). Postmortem examinations revealed an increase in the weight of the adrenal glands and heart, and a decrease in the weight of the thymus; histopathologically, there was an increase in degenerative changes in individual cells within the thymus. No changes in biochemical or hematological parameters were observed [71].

In a much earlier study, 12-day-old male mice of the B6C3F1 strain were administered α- or β-asarone—as a single dose (52 mg/kg, i.p.) or multiple doses (total dose approximately 1 mg/kg, intraperitoneally; administered on days 1, 8, 15 and 22); the development of hepatocellular carcinoma was observed after 10 and 13 months, respectively [72]. Other older data come from a study in which chicken embryos were exposed to β-asarone and a 100% mortality rate was observed, indicating the teratogenic properties of the compound under investigation [73].

Another point worth mentioning is that the oral bioavailability of β-asarone is low, and it has a short plasma half-life. On the other hand, due to its high lipophilicity, the compound under investigation is distributed quite well to organs with a high degree of perfusion, particularly the brain. Lower doses of this compound (>50 mg/kg) exhibit a broad range of pharmacological activity, which explains the positive effects observed in the context of potential AD therapy [71].

Data on the toxicity of tenuigenin as a pure compound are limited. There are publications describing potential adverse effects and safety studies of herbal preparations; however, it should be borne in mind that their potential effects, including toxic effects, may be associated with the presence of co-existing compounds, rather than tenuigenin itself. Wen et al. [74] demonstrated that tenuigenin contributed to a reduction in prostaglandin E2 concentrations in the stomachs of mice following administration of this compound at a dose of 200 mg/kg. Bearing in mind the fact that prostaglandin E2 performs a physiological protective and regenerative function for the gastric mucosa, its deficiency may result in gastric damage [74].

The bioavailability of tenuigenin following its oral administration, as compared with β-asarone, is also low, and it is, moreover, rapidly eliminated from the body. It should be borne in mind, however, that low bioavailability does not always imply the absence of toxicity or low toxicity. This type of ‘effect’ may lead to higher doses or more frequent administration, in order to enhance the therapeutic effect, and this alters the exposure profile [75].

In summary, the available evidence from pre-clinical studies and the limited clinical data suggest that multi-pronged approaches may be worth further investigation, both in terms of therapeutic effects and potential toxicity.

Potential drugs for the treatment of AD are shown in Table 1.

## 6. Potential Drugs for the Management Treatment of AD, Based on Preclinical Data

### 6.1. JQ1 (An Inhibitor of Bromodomain-Containing Protein 4)

Chronic JQ1 was administered intracerebroventricularly to rats also receiving Aβ, and, subsequently, the rats were tested in the Morris Water Maze [76], evaluating spatial memory and learning. JQ1 significantly shortened escape latency, and extended the time spent in the target quadrant. Also, JQ1 was responsible for increasing M2/M1 microglia polarization and the ratio of mature BDNF/proBDNF in the hippocampus. These results may indicate that JQ1 significantly improved learning and memory, which probably resulted from the elevated hippocampal level of mature BDNF and suppression of inflammatory factors [76].

### 6.2. Novel Harmine Derivatives

Liu et al. [77] developed a series of harmine derivatives as potent and selective glycogen synthase 3β inhibitors. Noteworthy, this enzyme plays a key role in tau-protein phosphorylation [78] and. moreover, it is overexpressed in the brain tissue of AD patients, which correlated with the degree of cognitive impairment [79]. Among the series of tested harmine derivatives, compound 39a effectively inhibited tau-protein phosphorylation in Tau protein (P301L)293T cells with an ED50 of 0.06 ± 0.01 µM. This harmine derivative was also given to okadaic acid-pretreated C57BL/6J and 3xTg-AD mice as models of AD. In both models of AD, a reduction in cognitive impairment was observed, without affecting motor performance in mice [77].

### 6.3. Inhibitors of Carbonic Anhydrase Isoforms and Phosphodiesterase 5

Carbonic anhydrase and phosphodiesterase inhibitors have been considered as potential agents for the management of AD [80,81]. Costa et al. [82] designed and evaluated a series of compounds as dual inhibitors of carbonic anhydrase isoforms and phosphodiesterase 5. The most promising compound was compound 8a, potently inhibiting the enzymatic targets. When added to SH-SY5Y culture in concentrations up to 100 µM, it did not significantly affect cell viability. Compound 8a (12.5–100 µM) was also effective in protecting against the cytotoxic effects of Aβ. Interestingly, the reduction in oxidative stress by this compound was significantly greater compared to acetazolamide (an inhibitor of carbonic anhydrases) or sildenafil (an inhibitor of phosphodiesterase 5) at equivalent concentrations of 12.5–100 µM. Compound 8a was also effective after in vivo experiments. A clear-cut protection against Aβ-induced deficits in recognition and working memory was observed [82].

### 6.4. Gemfibrozil

The mouse model of AD used in this study was Microtubule-Associated Protein Tau MAPT/PS19 [83]. In this model, a mutant form of human tau protein (P301S) is present in neurons. After daily oral administration of gemfibrozil (a ligand of peroxisome proliferator receptor α {PPAR α}), a reduction in the total tau protein and its phosphorylated form was observed in the hippocampal areas of CA1 and dentate gyrus. In addition, this treatment resulted in increased PPARα expression in the hippocampus, which was associated with a reduction in micro- and astrogliosis processes. However, in PPARα-deficient mice, gemfibrozil was ineffective against tau-protein pathology and gliosis. Finally, this drug was also effective in reversing cognitive impairment in MAPT mice during the symptomatic phase [83].

### 6.5. Coumarin Derivatives—Imperatorin and Umbelliferone

Coumarins, documented to exert anti-inflammatory and antioxidative effects [84], may bear an anti-AD potential. Recently, coumarin derivatives, imperatorin and umbelliferone, were tested in the rat scopolamine model [85] leading to cognitive impairment. Finally, their influence on neuroinflammation, oxidative stress, cholinergic dysfunction and metabolites of the kynurenine pathway were evaluated. Scopolamine was given once daily at 1 mg/kg, umbeliferone was administered per os once daily at 30 mg/kg, and imperatorinalso once daily per os, at 10 mg/kg. A Y-maze test (evaluating short-term spatial memory and locomotion) was conducted on day 8, a novel-object recognition test (short- and long-term memory) on days 9–11, and a passive-avoidance test (memory retention) on days 12–14. Scopolamine was injected 1 or 2 h, and coumarin derivatives 30 min or 1 h, prior to behavioral testing. Some behavioral tests were also conducted 24 h after the administration of coumarins. Evidently, both coumarins increased the rate of spontaneous alternations, as compared with the scopolamine group in the Y-maze test. As regards the novel-object recognition test, 30 min or 24 h after administration of coumarins, no significant effects in the rate of contacts were observed in pretreated animals’ vs. the scopolamine group. Only 60 min after administration of imperatorin, a significant increase in the rate of contacts was observed, vs. the scopolamine group. In the passive-avoidance test, both coumarins given 1 h before the test significantly increased the latency index vs. the scopolamine group. No significances were observed after 24 h. In addition to behavioral tests, umbeliferone reduced oxidative stress, inhibited acetylcholinesterase (AChE) activity and protected mitochondrial integrity. On the other hand, imperatorin reduced neuronal apoptosis and influenced cytokine levels, towards an anti-inflammatory profile [85].

### 6.6. Ponicidin

The 5 × FAD transgenic mice (3 months old, AD model) were treated with ponicidin for 4 weeks [86]. Then, a battery of behavioral tests was used (T-maze, Y-maze, Barnes maze and the Morris water maze) to assess learning and memory function. Vehicle pretreated 5 × FAD mice showed significant spatial memory deficits in the T-maze and Y-maze tests when compared to age-matched wild-type littermates. Also, in the Barnes maze, 5 × FAD mice exhibited reduced spatial orientation. Ponicidin (10 mg/kg) clearly prevented cognitive impairment. In the Morris water maze, the transgenic mice were characterized by, for instance, longer escape latencies or shorter dwell time, which was indicative of considerable cognitive deficits. In this test, ponicidin (10 mg/kg) also restored cognitive function.

Studies on the interaction of ponicidin with receptor-interacting protein kinase 1 (RIPK1) involved in neuroinflammation and necroptosis [87] were conducted with the use of bio-layer interferometry and drug affinity responsive target stability assays. It turned out that ponicidin bound RIPK1 with high affinity and a KD value of 135 nM and increased the resistance of RIPK1 to proteolytic degradation. In vitro effects of ponicidin were studied in microglial cells (BV2) and neurons (hippocampal HT22 cells). Evidently, ponicidin reduced the release of proinflammatory cytokines and tumor necrosis factor α (TNF-α), and this particular effect resulted from disrupting RIPK1-janus kinase 1 (JAK 1)-signal transducer and activator of transcription 1 (STAT 1) pathway. Also, by blocking RIPK1-RIPK3-mixed lineage kinase domain-like protein (MLKL), ponicidin reduced necroptosis mediated by RIPK1 in hippocampal neurons [86].

### 6.7. Salicin

Salicin (extracted from willow bark) was studied on cognitive performance in a scopolamine model of AD in mice, as well as on scopolamine-induced neurodegeneration in the cortex and hippocampus [88]. Recognition and spatial memory were evaluated in the novel-object recognition test and radial-arm maze test, respectively. The results clearly indicate that salicin significantly improved behavioral test scores, reducing learning errors and improving memory. A significant neuroprotective effect was also observed in the cortex and hippocampus. Analysis in silico suggests that the beneficial effects of salicin might be mediated via the neurotrophin-signaling pathway, glycerolipid-metabolism pathway, and cholinergic neurotransmission [88].

### 6.8. Calcaratarin D

Calcaratarin D is a landane-type diterpenoid obtained from the rhizome Hedychium flavum. It is characterized by a strong anti-inflammatory activity [89]. Its neuroprotective and procognitive effects were evaluated in a mouse model of AD induced by intrahippocampal administration of Aβ1-42 oligomers [90]. Calcaratarin D was given once daily for 14 days after the administration of Aβ1-42 oligomers. The following behavioral tests were included: the Morris water maze and novel-object recognition test. The AD mice exhibited significant learning and memory deficits, which were potently reduced by calcaratarin D pretreatment in the entire dose range. Also, in the novel-object recognition test, the performance of mice (potently reduced exploratory behavior) was significantly improved after administration of the diterpenoid. The results of lipidomic metabolomic analysis provided evidence that calcaratarin D partially reversed metabolomic dysregulation in AD mice. In particular, the modulatory influence of calcaraatrin D on sphingolipids and glycerophospholipids was observed. Finally, calcaratarin D decreased ceramide levels, and data from molecular docking indicate that this effect may be due to targeting ceramide transfer protein by the diterpenoid, which is responsible for its neuroprotective activity in AD mice. In in vitro experiments on BV2 cells, the diterpenoid (at concentrations of 5–20 µM) did not show any cytotoxic effects. Moreover, calcaratarin D (in the entire concentration range) significantly reduced TNF-α, IL-6 and IL-1β levels elevated by lipopolysaccharide (LPS) + Aβ. A series of further experiments on the involvement of signaling pathways in the beneficial effects of the diterpenoid revealed that calcaratarin D blocked the TLR4/NF-κB/NLRP3 axis [90].

### 6.9. New 1,3,5-Triazines

A number of triazine compounds were synthesized that exhibited nanomolar affinity for 5-HT6, 5-HT2A, and D2 receptors. These compounds also inhibited fatty-acid amide hydrolase and exerted antioxidant effects [91]. In cellular models of AD pathologies, a number of compounds exerted beneficial effects on mitochondrial dysfunction and toxicity induced by A-β and glutamate. The thio-analogue, designated as compound 4c, crossed the blood–brain barrier well, and at 1 and 3 mg/kg effectively reversed MK-801-induced memory deficit in the novel-object recognition test in rats [91].

### 6.10. Novel 2-Aminopyrymidine Carboxamide Derivatives

A series of 2-aminopyrimidine-carboxamide derivatives demonstrated AChE inhibitory activity, while being significantly less effective against butyrylcholinesterase [82]. Compound 9s is particularly noteworthy, as it exhibited stronger AChE inhibition (IC50 = 1.60 µM) than the positive control, galantamine (IC50 = 5.10 µM). In the DPPH (2,2-diphenyl-1-picrylhydrazyl—a stable free radical) radical-scavenging assay, most compounds showed weak antioxidant activity. However, compound 9p displayed relatively high activity (IC50 = 113.9 µM), although this value was still higher than that of ascorbic acid (IC50 = 41.17 µM), the positive control. To date, no in vivo data are available [92].

### 6.11. Epimedium Brevicornu Flavonoids

Epimedium brevicornu flavonoids (EFs) were studied in the LPS model of AD in 8–9-month-old C57BL/6J mice [93]. In the freeze-dried EF extract, the main ingredients are Epimedin A, B, C, and icariin. Also, icariside II (another flavonoid) was added, as an agent with strong neuroprotective properties.

The extract was given orally, at 50–200 mg/kg, for 14 days and then, on day 15th, LPS was administered. Administration of EFs was continued until day 21. Mice, after 8 h following LPS, exhibited a reduced number of crossings and rearings in the open-field test, clearly indicating a decline in locomotor and exploratory activity. The extract in the whole range of doses reversed locomotor deficit without affecting, however, the impaired exploratory activity. Positive controls with donepezil (1.5 mg/kg orally) and ibuprofen (50 mg/kg orally, both drugs given for 21 days) did not show any beneficial effect in this test.

On day 16th, the Y-maze test was carried out. Mice administered LPS showed marked impairment of working memory, manifested by a considerable reduction in the number of arm entries and spontaneous alternation. At the highest dose, EFs inhibited LPS-induced working-memory deficits, whereas at the intermediate dose it only affected spontaneous alternation.

Long-term spatial memory was evaluated in the Morris water maze test (escape-acquisition phase) on days 17–19. The LPS group demonstrated much longer escape latencies and slower learning, as compare to the control animals. The EF-treated group exhibited a significant reduction in escape latency throughout the learning period, which was a clear indicator of improved spatial learning. Ibuprofen, as a positive control, showed a comparative effect to that of EFs. In contrast, donepezil had no effect on the spatial-memory deficit induced by LPS. Once the escape-acquisition phase was completed, the mice were subjected to a probe test on day 20. Mice exposed to LPS exhibited typical spatial-memory impairments, which manifested as less time spent in the target quadrant and fewer platform crossings, in comparison to the control group. EF treatment significantly improved the performance of mice in this test, and the behavior of animals administered the highest dose of EPs (200 mg/kg) did not differ from that of the control group.

The animals were sacrificed on day 21, and a series of biochemical estimations were performed. Administration of LPS resulted in a significant elevation of serum and hippocampal levels of pro-inflammatory cytokines (IL-6, IL-1β, TNF-α). On the other hand, serum concentrations of anti-inflammatory cytokines (IL-4, IL-10) were distinctly reduced by LPS. Pretreatment with EF dose-dependently inhibited the negative effect of LPS upon cytokine concentrations, both in the serum and hippocampus. To delineate a possible effect of EFs upon Aβ pathology, the concentrations of APP, BACE1, and Aβ42 in the hippocampus and cortex were evaluated. Following LPS administration, an increase in Aβ42 levels was observed in the cerebral cortex and hippocampus. BACE1 expression in the cerebral cortex also increased. However, APP levels remained unchanged. In the presence of EFs at doses of 100 and 200 mg/kg, no increase in Aβ42 levels was observed in the cerebral cortex and hippocampus. Furthermore, a decrease in BACE1 expression was noted in the cortex. Interestingly, EFs (100 and 200 mg/kg) reduced the cortical APP concentration. Regarding cortical tau-protein level, LPS caused an increase in the concentration of phosphorylated tau protein, and EFs (100 and 200 mg/kg) significantly prevented this negative effect.

The anti-inflammatory activity of EFs was studied by the expression of cortical and hippocampal Iba1 (a microglial marker) and GFAP (an astrocytic marker). Both markers were markedly elevated, as a result of exposure to LPS, along with morphological changes such as microglial hypertrophy and retraction of astrocytic processes. Treatment with EFs dose-dependently prevented all above-mentioned alterations. Also, positive controls (ibuprofen and donepezil) exerted comparable anti-inflammatory effects.

EFs also exhibited neuroprotective properties. In a dose-dependent manner, clear-cut neuroprotection was provided against LPS-induced profound neuronal loss in the hippocampal CA3 area. It is very likely that the anti-inflammatory and neuroprotective activity of EFs may result from the attenuation of stimulatory LPS effect on PI3K/AKT and cGAS-STING signaling pathways [93].

One of the EFs, icariin, was evaluated individually in the Aβ model in rodents [94,95,96]. Rats were administered icariin for 28 days. The treatment effectively reversed spatial-memory deficits in the Morris water maze test. Moreover, the flavonoid reduced Aβ accumulation, counteracted Aβ-induced activation of autophagy, ameliorated mitochondrial and nuclear damage in the hippocampus, and mitigated brain oxidative stress and astroglia activation. Other studies confirm the beneficial effects of icariin in the Aβ model in mice [95,96].

### 6.12. The Glymphatic System—A Target for Potential Anti-AD Drugs?

The glial lymphatic system, together with the meninges, constitutes the cerebral lymphatic system [97]. The glymphatic system is a “brain cleansing” system, based on a perivascular pathway for the exchange of cerebrospinal fluid (CSF) with interstitial fluid (ISF). This process naturally supports the homeostasis of brain cells, and removes harmful byproducts such as neurotoxic proteins—including amyloid aggregates, α-synuclein, tau protein, and metabolic byproducts [98,99,100]. Structurally, the glymphatic system consists of three components: the arteriovenous perivascular spaces—the gaps between the vessel wall and astrocytic end-feet (the main channel), aquaporin-4 (AQP4) channels, and the ISF compartment—a site where brain metabolites, including Aβ aggregates, accumulate [97,101]. It has been shown that the activity of this system is strictly regulated by the cardiovascular system, and the flow is generated by the pulsation of arterial walls, which is linked to the cardiac cycle. This mechanism allows CSF to flow around the blood vessels into the brain interstitium, and then drain into the venous system [99]. Research on this process suggests that dysfunction of this system may be a common factor in the pathophysiology of many nervous system diseases, including those involving vascular, inflammatory, or astroglia disorders, among others. A link has also been found with conditions such as epilepsy, headaches of various origins, AD, Parkinson’s disease, multiple sclerosis, and idiopathic intracranial hypertension [98].

AQP4, a water channel located primarily in the processes of astrocytes, plays a key role in the functioning of the glymphatic system [98]. Studies using animal models have shown that the absence of AQP4 or its abnormal localization, by impairing CSF flow, has been directly linked to reduced clearance of tau protein and β-amyloid peptides, thereby contributing to the development of AD symptoms [102]. Lopes et al. [103] set out to evaluate the relationship between the glymphatic system and the deleterious effects of tau protein by employing long-term modulation of lymphatic function. To this end, they used chronic administration of TGN-020—an AQP4 inhibitor—on Thy1-hTau protein. P301S mice were intracerebrally inoculated with tau protein. In the study group, a higher degree of tau-protein aggregation and spread in the brain was observed, as well as more severe brain atrophy. Furthermore, the treated mice exhibited more severe cognitive impairments, as assessed in behavioral tests [103]. In a transgenic mouse model of Tet-Off APP amyloidosis, the authors evaluated the function of the glymphatic system. The Gd-DOTA contrast agent was used for the assessment; it was injected into the cisterna magna, and its transport throughout the brain was then monitored using contrast-enhanced MRI (DCE-MRI). Additionally, “region-of-interest analysis and computational modeling were used to assess GS dynamics, while characterization of brain tissue abnormalities at the microscale was performed ex vivo by immunohistochemistry.” The results unequivocally indicated impaired lymphatic function and a high burden of amyloid plaques in the experimental group, as well as extensive astrogliosis and microgliosis, and a neuroinflammatory response [104]. Another study demonstrated that the gene signature of microglia derived from 5 × FAD mice with impaired meningeal-lymphatic function overlaps with the transcriptional profile of activated microglia in patients with AD. These results also confirm that impaired glymphatic-system function promotes the development of microglial inflammation, which is characteristic of AD [105].

There are also reports indicating a significant association between patients carrying the APP, PS1, or PS2 genes and AQP4 depolarization, and thus glymphatic-system dysfunction [106].

Furthermore, this process is most efficient during sleep, when the interstitial space expands, and this may also, to some extent, explain nervous system dysfunction under conditions of prolonged sleep deprivation [107,108,109]. Holth et al. [109] demonstrated that the sleep–wake cycle regulates Aβ levels in interstitial fluid and cerebrospinal fluid. Studies conducted on mice showed that chronic sleep deprivation increased the number of Aβ plaques, as well as tau-protein levels in interstitial fluid and cerebrospinal fluid. In humans, tau-protein levels may increase by as much as 50% or more, in cases of chronic sleep deprivation. These findings point to another factor that can significantly increase the risk of developing AD.

#### 6.12.1. Oxytocin

The link between the glymphatic system and AD is a relatively new discovery—consequently, research into potential treatment methods is still lacking.

One of the proposed treatment methods is oxytocin. Oxytocin was administered intranasally to middle-aged APP/PS1 mice at a volume of 20 μL every other day, a total of 15 times. The control group consisted of APP/PS1 mice and age-matched C57BL/6J mice that received saline, forming the AD group and the wild-type group, respectively [110]. To assess intracranial lymphatic drainage, the authors used cognitive behavioral tests, in vivo imaging of live mice, in vivo two-photon imaging, ASL/CEST-MRI scanning, immunofluorescent staining, ELISA, RT-qPCR, Western blotting, and RNA-seq analysis. Following the experiment, the authors demonstrated that exogenous oxytocin improves the function of both the glymphatic and meningeal systems, and also contributes to the normalization of brain structure. Consequently, effective clearance of Aβ from the brain into the cerebrospinal fluid and, subsequently, to the deep cervical lymph nodes was observed. Furthermore, improvements were observed in cerebral hemodynamics, AQP4 polarization, meningeal lymphangiogenesis, and transcriptional profiles. Finally, behavioral tests revealed the restoration of cognitive function in a mouse model of AD [110].

As of today, there are no available results from clinical trials assessing the effect of oxytocin therapy in patients with AD. However, it is worth noting that there is evidence suggesting that oxytocin levels in patients with AD are significantly lower than in healthy individuals. Furthermore, there is a positive correlation between the volume of the right parahippocampal gyrus and plasma-oxytocin concentration, and a negative correlation between its volume and oxytocin-signal intensity in these patients. This suggests that, in AD, oxytocin-related abnormalities may involve brain regions critical for cognitive processes [111,112]. Similar conclusions were reached by Zou et al. [103], who analyzed gene expression profiles obtained from the GSE63060 (for AD) and GSE162790 (for cerebral small-vessel disease) databases. A comparative analysis of the data revealed the presence of 146 genes with differential expression, belonging to the oxytocin-signaling pathway, which confirms the need for further research into potential therapies [113].

#### 6.12.2. Anti-CD25 Antibodies

Da Mesquita et al. [114] based their study on the fact that the aging process contributes to the deterioration of the lymphatic vessels of the meninges and peripheral immune system, which, in turn, significantly accelerates the decline in cognitive function. They suggested the existence of an age-related mechanism in which there is a reduction in the efflux of immune cells through the lymphatic system, dependent on the chemokine receptor type 7 (CCR7). Immune dysfunction in the meninges associated with reduced CCR7 expression contributes to changes in the function of astrocytes and microglia in the aging brain, which, in turn, affects the lymphatic system, the accumulation of Aβ in the brain, and the decline in cognitive function.

The experiment used older (25-month-old) male C57BL/6J mice, which were then genetically crossed to produce mice that were heterozygous for CCR7GFP or deficient in CCR7 (CCR7−/−) from a non-transgenic or 5 × FAD background (hemizygous for the transgene) [114]. As a result, the animals represented a transgenic mouse model of familial AD. The control group consisted of mice from the same litter and of the same age. In practice, reduced CCR7 expression by meningeal T cells in older mice is associated with an increase in the number of effector and regulatory T cells, while CCR7 deficiency in the hematopoietic system mimicked the age-related changes in meningeal T cells, which in turn led to reduced cerebrospinal fluid flow and cognitive impairment. 5 × FAD transgenic mice with CCR7 deletion exhibited harmful activation of the neurovascular system and microglia, as well as increased Aβ accumulation in brain tissue. The test mice were administered rat IgG1 antibodies (total 250 μg; clone HRPN, BE0088, Bio X Cell) or anti-CD25 antibodies (total 250 μg; clone PC-61.5.3, BE0012, Bio X Cell) intraperitoneally. One week later, after the Morris water maze test and before the open-field test and the novel-location recognition test, the antibodies were re-administered. Immunochemical analyses showed that anti-CD25 antibodies attenuated the enhanced response of regulatory T cells in the meninges and lymph nodes, while behavioral tests demonstrated improved cognitive function in aged mice [114].

#### 6.12.3. Borneol

Borneol (C10H18O) is a natural, herbal compound that is highly soluble in lipids and belongs to the group of bicyclic monoterpenes. It crosses the blood–brain barrier very rapidly (within 5 min of oral administration, reaching peak concentration in the brain within 1 h) [115]. According to studies using mouse models of cerebral ischemia, borneol (administered via a gastric tube at 200 mg/kg, 3 days before inducing ischemia) combined with mesenchymal stem cells (injected intravenously 24 h after ischemia) significantly reduced the area of neuronal necrosis and cell apoptosis, enhanced neurogenesis, and improved functional recovery, compared to the control group which received mesenchymal stem cells alone [115]. Furthermore, the literature indicates that the compound under study possesses neuroprotective, anti-inflammatory, and antiepileptic properties [116,117,118,119]. Furthermore, based on studies of borneol in animal models of various neurological diseases, mechanisms such as a reduction in acetylcholinesterase activity, enhanced cholinergic transmission in the brain, and increased concentrations of vascular endothelial growth factor have been demonstrated [120,121].

Wu et al. [122] evaluated borneol in a mouse model of AD. To obtain results regarding both changes in the glymphatic system and behavior, the study employed techniques such as immunofluorescence staining, Western blotting, ELISA, RT-qPCR, and nitric oxide assay kits. Behavioral tests included the open-field test and the Morris water maze. Borneol increased lymphatic drainage by improving permeability and increasing the internal diameter of lymphatic vessels, which in turn accelerated the removal of Aβ from the brain. On the other hand, by reducing nitric oxide levels in the meninges, borneol stimulated lymphatic vasoconstriction and promoted lymphangiogenesis, by increasing levels of FOXC2, vascular endothelial growth factor C (VEGFC), and LYVE-1 in the meninges, which in turn accelerated the clearance of macromolecules. Improved lymphatic clearance in the meninges was also demonstrated, which promoted not only the removal of Aβ, but also other harmful macromolecular polymers. From a behavioral perspective, borneol also significantly alleviated cognitive deficits [122].

Results of preclinical studies may be found in Table 2.

## 7. Drug Combinations in AD

Since AD is a multifactorial disorder, the use of pharmacological combination therapy appears to be justified. Furthermore, this is supported by the fact that therapies targeting a single pathology (e.g., amyloid plaques) slow down cognitive impairment by 30%, while having no effect on the remaining 70% [123].

Combinations can be of an “add-on” nature, where a second drug is incorporated into an existing single-drug therapy; an example of this is the addition of memantine to donepezil, which often yields positive clinical outcomes regarding cognitive function. In the context of potential anti-AD treatments, it is particularly relevant to employ combinations of two drugs that target different mechanisms involved in the pathogenesis of AD, simultaneously—for instance, combining anti-tau agents with novel neuroprotective or anti-inflammatory drugs. Naturally, the “add-on” approach can also be considered for experimental or potential therapeutic agents. Furthermore, such combinations may incorporate agents designed to enhance blood–brain barrier penetration (e.g., trontinemab). A distinct type of combination involves the use of agents with a complex, multi-faceted mechanism of action—effectively functioning as a “combination in a pill.” An example of this is posiphen, which, in addition to reducing the APP—as previously mentioned—can also decrease levels of tau protein, α-synuclein, and other toxic proteins, by inhibiting mRNA translation [123].

Naturally, combination therapies are used in animal models of AD, where toxic effects can be assessed, and dosing guidelines for phase 1 clinical trials can be developed. If the components of a combination are entirely new agents, the FDA requires an evaluation of the combination’s effects across various parameters, such as absorption, distribution, metabolism, excretion, toxicity, and carcinogenicity. The topic of drug combinations in AD is discussed extensively in an article by Cummings et al. [123].

## 8. Conclusions and Future Prospects

It is a completely obvious fact that most existing pharmacological therapies provide only temporary symptomatic relief, without a significant impact on the progression of AD [124]. Therefore, the search continues for drugs that, in addition to providing symptomatic relief, would inhibit the mechanisms driving the development and progression of AD. Completely new compounds that show promising activity in animal models are being intensively evaluated. Some of these have been presented above, and preliminary clinical data already exist for some of them. Typically, compounds that work in animal models of AD also showed some positive effects in patients in a number of tests assessing, for example, memory and cognitive abilities. In any case, their clinical results are not particularly encouraging.

Moreover, there are cases where this relationship between animal and clinical research did not exist, or was limited. An example would be 5-HT6 receptor antagonists, most of which have not shown any positive effects in clinical settings.

There are many compounds that show promising activity in animal models of AD, and perhaps some of them will be clinically evaluated. The compounds shown above exhibit various mechanisms of action, such as, for example, limiting neuroinflammation, oxidative stress and cholinergic dysfunction. Of course, many more substances with potential anti-AD effects are currently being evaluated, and they have many other mechanisms of action. Most studies concern single substances, and it turns out that the influence on a given signaling pathway may determine a positive effect in models of this disease. As mentioned above, encouraging results from experimental studies do not translate into clinical outcomes. As shown in Table 3, only posiphen (buntanetap) shows promise for use in AD, although it did not yield positive results in all studies. However, the small number of patients may complicate the assessment, especially in the case of negative results. Perhaps the positive result of the randomized (blinded and placebo controlled) study was due to a much larger number of patients, longer use, and a significantly smaller dose (15 mg per day, compared to 80 and 60 or 120 mg per day). One can also have hope regarding ACD856, as it is being prepared for phase 2 studies.

The other medications failed to meet their endpoints. It is also worth noting that some potential anti-AD drugs are being evaluated for other neurodegenerative diseases, as well (Table 3).

For the majority of potential anti-AD drugs undergoing clinical evaluation, randomized, blinded, placebo-controlled trials—ranging from phase 1 to phase 3—are available. These studies offer the highest level of reliability. A pertinent example is bezisterim, for which open-label study results are also available; such studies typically lack a placebo group, and are not blinded. It was found that an open-label trial involving 23 patients provided evidence of a positive treatment effect across a number of assessment scales. However, a randomized trial conducted on a significantly larger group of patients failed to confirm these effects. The other medications failed to meet their endpoints in reliable randomized trials. It is also worth noting that some potential anti-AD drugs are being evaluated for other neurodegenerative diseases, as well (Table 3).

As mentioned in Section 7, to achieve the desired clinical effects, multitargeted therapies might be used. An example of such a strategy is the combination of memantine with beta-asarone and tenuigenin presented above, which significantly improved the patients’ scores on the MMSE, ADL and CDR scales, without increasing the side effects. Moreover, combinations between already registered drugs are also used, e.g., memantine and donepezil. This combination had a significantly better effect in patients with severe cognitive impairment compared to donepezil alone, as shown by SIB (severe impairment battery) scores [21]. This study, conducted using the meta-analysis method, showed, however, a large heterogeneity between the analyzed results and, moreover, no significant differences were found in relation to MMSE scores. The available preclinical and limited clinical evidence suggests that multitargeted approaches may warrant further investigation.

This article focuses on reducing cognitive deficits through pharmacological intervention. Naturally, neuropsychiatric symptoms also represent a significant issue in AD. Atypical antipsychotic drugs may be considered, although their efficacy is moderate and their safety profile is poor [135]. Therefore, personalized psychosocial interventions should be considered first and foremost, as they help reduce agitation and symptoms of depression. Newer medications can be used for agitation (brexiprazole) and psychosis (pimavanserin). However, their effect sizes and safety profiles require careful interpretation [135]. Interestingly, the aforementioned masupirdine actually reduced agitation/aggression symptoms in AD patients, as shown in a phase 2 clinical trial. A phase 3 trial is in progress [136]. A full description of the approach to neuropsychiatric symptoms requires a separate article.

Future clinical evaluation of potential anti-AD drugs or their combinations requires confirmation of the diagnosis—for example, by demonstrating amyloid pathology (e.g., via PET) or the presence of phosphorylated tau 217 protein in the plasma. In addition to its diagnostic value, phosphorylated tau 217 protein may also serve as a prognostic biomarker, as high concentrations are associated with a greater likelihood of progression from AD-associated mild cognitive impairment to AD dementia. Clinical testing of new compounds—or combinations, thereof, in such patients—against a placebo group, could reveal their beneficial effects. There are other types of biomarkers in AD; for example, predictive biomarkers, target engagement biomarkers, disease impact pharmacodynamic biomarkers, or safety biomarkers. The identification of biomarkers is essential for planning clinical trials in which different groups of patients—for example, those with varying levels of the tau protein or *APOE4* gene carriers vs. non-carriers—can be compared. Furthermore, the analysis of biomarker levels can be used to evaluate the efficacy of the drugs (or drug combinations) being studied [123].

Regarding the glymphatic system, a significant portion of the evidence comes from animal models. As for oxytocin, there is a clear lack of clinical trial results involving patients with Alzheimer’s disease. It must therefore be stated unequivocally that any therapeutic hypotheses related to this system are currently at a preclinical stage, and do not yet represent available treatment options.

As previously mentioned, episodes of cerebral ischemia can lead to Aβ accumulation, tau-protein modification, neurodegeneration, and cognitive impairment. It is also worth noting that episodes of cerebral ischemia were associated with the dysregulation of a number of genes responsible for APP and tau-protein metabolism, as well as the processes of autophagy, mitophagy, and apoptosis. It cannot be ruled out that preventing the dysregulation of the aforementioned genes could become a future preventive strategy for AD [137].

Present and future therapies for AD may be characterized by low bioavailability and poor targeting, due to the blood–brain barrier. This can be overcome by nanoparticle-based drug delivery systems. A variety of nanoparticles are available, such as liposomes, exosomes, dendrimers, and carbon dots. Preclinical studies clearly point to their favorable biological safety profile and marginal cytotoxicity. However, their widespread use must be preceded by studies of their functional toxicity, potential excitotoxicity and DNA damage [138].

## Figures and Tables

**Table 1:** Potential drugs for the treatment of Alzheimer’s disease—clinical data.

Drugs (Clinical Trial, Phase) Clinical Trial ID		Mechanism of Action	Patients		Dosage			Scales Used				Additional Data	Safety/Adverse Events	Ref.
			Age	Diagnosis	Dose [mg]	Method of Administration	Duration	Results						
Meta-analysis	Semorinemab (Lauriet study included a randomized, placebo-controlled, double-blind period, phase 2) NCT03828747	Anti-tau monoclonal antibody.	Age: 50–85 years.	Mild-to-moderate AD.	4500	IV	Every 4 weeks for 48 or 60 weeks.	ADAS-Cog11	ADCS-ADL	MMSE	CDR-SB	n/d	Safe and well-tolerated.	[25,26]
								↓	0	0	0			
	Semorinemab (randomized, double-blind, placebo-controlled, parallel-group clinical trial, phase 2) NCT03289143		Mean age, 69.6 years.	Prodromal or mild AD.	1500 or 4500 or 8100	IV	Every 2 weeks for the first 3 infusions and every 4 weeks thereafter.	-	-	-	CDR-SB	n/d	Safe and well-tolerated.	[25,27]
											0			
	Zagotenemab (randomized, double-blind, placebo-controlled phase2 study) NCT03518073	Anti-tau monoclonal antibody	Mean age = 75.4 years.	Early symptomatic AD.	1400 or 5600	IV	Every 4 weeks for 100 weeks.	IADRS	-	-	-	A dose-related increase in plasma tau.	Current side effect.	[25,29]
								0						
	Zagotenemab (multi-site, participant- and investigator-blind, placebo-controlled, parallel-group study, phase 1B) NCT03019536		Age: 59 to 84 years.	Mild cognitive impairment due to AD or mild-to-moderate AD.	70 or 210	IV	Every 4 weeks for up to 49 weeks, followed up for 16 weeks.	-	-	-	-	Increase in plasma tau.	4 serious adverse events occurred in 2 participants.	[25,30]
	Tilavonemab (randomized, double-blind, placebo-controlled study, phase 2) NCT02880956	Anti-tau monoclonal antibody.	Aged 55–85 years.	Early-AD disease.	300 or 1000 or 2000	IV	Every 4 weeks for 96 weeks.	ADAS-Cog11	ADCS-MCI-ADL-24	MMSE	CDR-SB	Positive effect on hippocampal volume at higher dose levels.	Safe and well-tolerated.	[25,28]
								0	0	0	↓			
Fosgonimeton (randomized, double-blind, placebo-controlled phase 1 trial) NCT03298672		Highly specific, small-molecule positive modulator of the HGF/MET neurotrophic system.	Aged 18–45 years.	Healthy	Single ascending dose: 2, 6, 20, 40, 60, or 90.	IV	For 1 day.	TMT A&B test	-	MMSE	-	Enhancement of synaptic function and potential procognitive effects.	Safe and well-tolerated.	[40]
								0		0				
Fosgonimeton (randomized, double-blind, placebo-controlled phase 1 trial)			Aged 60–85 years.	Healthy	Multiple ascending dose: 20, 40, 60 or 80.	IV	For 9 days.	TMT A&B test	-	MMSE	-			
								0		0				
Fosgonimeton (randomized, double-blind, placebo-controlled trial, phase 2)			Aged 60–85 years.	Mild-to-moderate-AD patients.	40 or 70 once daily.	Subcutaneous	For 26 weeks.	ADAS-Cog11	-	-	-	Normalization of ERP P300 latency.	Safe and well-tolerated.	[41]
								↑	-	-	-			
Blarcamesine (randomized, placebo controlled, clinical trial, phase 2B/3) NCT04314934		A sigma-1 receptor agonist.	Aged 60–85 years.	Early AD	30 (*N* = 338) or 50 (*N* = 170) once daily.	Orally	For 48 weeks.	ADAS-Cog13	ADCS-ADL	-	CDR-SB	Decrease in brain volume loss; increase in plasma Aβ42/40-ratio.	Treatment-related serious adverse effects were recorded in 16.7% patients.	[44]
								↑	0	-	↑			
Bezisterim (NE3107) (open-label study, phase 2 trial) NCT05227820		Inhibitor of TLR4- and tumor necrosis factor-induced activation of NF-κB and reducer of ERK phosphorylation.	Aged 50–89 years.	Mild cognitive impairment or mild dementia.	20 twice daily.	Orally	For 3 months.	ADAS-Cog11	GRC	MMSE	CDR	A reduction in the levels of TNF-α, beta-amyloid and phosphorylated tau protein.	Safe and well-tolerated.	[48]
								↑	↑	↑ (no statistical significance)	↑			
Bezisterim (NE3107) (double-blind, randomized, placebo-controlled, parallel group, multicenter phase 3 trial) NCT04669028			Aged 60–85 years.	Mild-to-moderate probable AD.	20	Orally	For 30 weeks.	ADAS2 Cog12	GST	MMSE	CDR-SB	n/d	Side effects insignificant compared to placebo.	[49]
								0	0	0	0			
Posiphen (Buntanetab) (Double-blind phase 1b randomized clinical trial) NCT04524351		Positive enantiomer of (-)-phenserine.	Aged 45–85 years.	Early AD	80	Orally	For 25 ± 2 days.	ADAS-Cog11	WAIS	-	-	Improvement in axonal integrity and synaptic function; reduction in the levels of neurotoxic proteins and inflammatory factors.	Safe and well-tolerated.	[53]
								↑	↑	-	-			
Posiphen (Buntanetab) (Double-blind phase 1b randomized ascending dose clinical trial) NCT02925650			Aged 55–89 years.	Mild cognitive impairment or mild AD.	60	Orally	Once or twice daily for 21–23 days.	ADAS-cog12	NPI	MMSE	-	Decrease in APP synthesis.	Safe and well-tolerated.	[54]
								0	0	0	-			
Triazinetrione derivative (ACD856) (Randomized, double-blind, placebo-controlled trial, phase 1) NCT05077631		Positive allosteric modulator of neurotrophin receptors.	Aged 18– < 65 years.	Healthy	Single dose of 1-to-150 mg.	Orally	One day	-	-	-	-	n/d	Safe and well-tolerated.	[58]
								-	-	-	-			
β-Asarone + tenuigenin		Tenuigenin—neuroprotective effect; protection of neurons against Aβ-induced damage/apoptosis β-asarone—neuroprotective effects; reduction in apoptosis, inflammatory response, and Aβ-related disorders.	Aged 60–85 years.	Moderate-to-severe AD.	20	Orally	For 12 weeks.	ADL	-	MMSE	CDR	n/d	Safe and well-tolerated.	[67]
								↑	-	↑	↑			
β-Asarone + tenuigenin			Aged 60–85 years.	Moderate-to-severe AD.	10	Orally		ADL	-	MMSE	CDR	n/d		[68]
								↑	-	↑	↑			

-—this scale was not used; ↓ decrease/deterioration compared to the control group/placebo.; ↑ increase/improvement compared to the control group/placebo; 0—no effect; ADAS-Cog—Alzheimer’s Disease Assessment Scale—Cognitive Subscale; ADCS-ADL—Alzheimer’s Disease Cooperative Study—Activities of Daily Living scale; ADCS-MCI-ADL-24—24-item Alzheimer’s Disease Cooperative Study/Activities of Daily Living Scale Adapted for Patients With Mild Cognitive Impairment; ADL—Activities of Daily Living, CDR—Clinical Dementia Rating Scale; CDR-SB—Clinical Dementia Rating–Sum of Boxes; ERK—extracellular signal regulated kinases; ERP P300—Event-related potential P300; GRC—Global Rating of Change; GST—Global Statistical Test of Cog12; IADRS—Integrated AD Rating Scale; IV—intravenous infusions; MMSE—Mini-Mental State Examination; n/d—no data; NPI—Neuropsychiatric inventory; TLR4—Toll-like receptor 4; TMT—Trail making test; TNF-α—tumor necrosis factor α; WAIS—Wechsler Adult Intelligence Scale.

**Table 2:** Potential drugs for the treatment of Alzheimer’s disease—data from animals studies.

Drug/Substance	Dosage	Method of Administration	Duration of Treatment	Experimental Model	Behavioral Tests	Behavioral Effects	Additional Effects	References
ACD856	3 mg/kg	Subcutaneous	A single administration.	C57BL/6J mice—scopolamine-induced neurodegeneration; age: 18 months.	Passive avoidance.	Improved memory after 11 days.	Potentiation of the cellular signaling of the neurotrophin receptors.	[56]
Anti-CD25 antibodies	250 μg	Intraperitoneally	Twice, one week apart.	25-month-old 5 × FAD transgenic mice with CCR7 deletion.	Morris water maze; open-field; novel-location recognition.	Improved cognitive function.	Attenuation of the pathological response of regulatory T cells in the meninges and lymph nodes.	[114]
β-Asarone	10 mg/kg	Intragastrically	For 30 days.	APP/PS1 transgenic mice.	Water maze; passive avoidance; step-down.	Improved learning and memory.	A decrease in acetylcholinesterase levels in the hippocampus and in Aβ42 levels. An increase in the expression of phosphorylated m-TOR and p62. A decrease in the expression of phosphorylated Akt, beclin-1, and LC3B.	[64]
β-Asarone	15, 30 or 60 mg/kg	Orally	For 8 weeks.	Nine-month-old 3xTg-AD mice.	Morris water maze.	Improved cognitive function.	Reduction of hippocampal neurodegeneration. Reduction of Aβ accumulation and excessive tau phosphorylation in neurons of the hippocampal CA3 region.	[65]
β-Asarone	30 mg/kg	No data	For 8 weeks.	3xTg-AD mice.	Morris water maze; step-down.	Improved learning and memory.	Reduction in neuron loss; Decreased expression of Aβ, APP, tau-protein phosphorylation and neuroinflammatory markers. Inhibition of NLRP3 components.	[66]
Blarcamesine	0.1–1 mg/kg	Intraperitoneally	For 7 days before intracerebroventricular administration of the amyloidogenic Aβ fragment.	Mice—Aβ-induced AD model.	Y-maze; passive avoidance.	Prevention of spatial working-memory disorders and long-term memory disorders (at a dose of 1 mg/kg).	Inhibition of lipid peroxidation in the hippocampus (at a dose of 1 mg/kg).	[43]
Borneol	20 mg/kg	Orally	For 14 days.	Mice—Aβ-induced AD model.	Open-field test; Morris water maze.	Improvement in cognitive function.	Increased lymphatic drainage by improving permeability and increased internal diameter of lymphatic vessels, which in turn accelerated the removal of Aβ from the brain; promoted lymphangiogenesis by increasing levels of FOXC2, VEGFC, and LYVE-1 in the meninges.	[122]
Calcaratarin D	2.5, 5 or 10 mg/kg	Intraperitoneally	Once daily for 14 days after the administration of Aβ1-42 oligomers.	C57BL/6J mice (mouse model of AD induced by intrahippocampal administration of Aβ1-42 oligomers).	Morris water maze; novel-object recognition.	Improved learning and memory; improvement in exploratory behavior.	Partial reversal of metabolomic dysregulation; increased levels of sphingolipids and glycerophospholipids; decreased levels of ceramide.	[90]
Compound 39a	5 or 25 mg/kg/daily	Orally	For 2 weeks.	Okadaic acid-pretreated C57BL/6J and 3xTg-AD mice.	Morris water maze; novel-place recognition; open-field.	Reduction in cognitive impairment, without affecting motor function.	Inhibited tau-protein phosphorylation; demonstrated favorable permeability of the blood–brain barrier.	[77]
Compound 8a	4 mg/kg daily, starting 7 days before ICV administration of Aβ.	Intraperitoneal	For 14 days, but the cognitive tests were performed on days 6–8 and 12–14.	Mice—Aβ-induced AD model.	Novel-object recognition; Y-maze spontaneous alternation.	A clear-cut protection against Aβ-induced deficits in recognition and working memory was observed.	Protective effects against Aβ-induced cytotoxicity (SH-Sy5Y cells).	[82]
Epimedium brevicornu flavonoids	50, 100 or 200 mg/kg	Orally	For 21 days, but on day 15, LPS was administered.	8–9-month-old C57BL/6J mice.	Open-field; Y-maze (day 16); Morris water maze (on days 17–19).	Reversed locomotor deficit without affecting, however, the impaired exploratory activity. Reduction inworking-memory deficits induced by LPS (at dose 200 mg/kg); improved spatial learning.	Decrease in the concentration of pro-inflammatory cytokines in serum and the hippocampus. Decrease in BACE1 expression in the cortex. Reduction in the cortical APP concentration. Prevention of an increase in pro-inflammatory markers in the cortex and hippocampus. Neuroprotective effect.	[93]
Icariin	30, 60 or 90 mg/kg	Orally	For 28 days	Rats—Aβ-induced AD model.	Morris water maze.	Reversed spatial-memory deficits.	Reduction in Aβ accumulation, counteracted Aβ-induced activation of autophagy, ameliorated mitochondrial and nuclear damage in the hippocampus. Mitigated brain oxidative stress and astroglia activation. Improvement in autophagy and lysosomal function.	[94]
JQ1	No data	Intracerebroventricularly	No data	Rats—Aβ-induced AD model.	Morris water maze.	Improved learning and memory.	Increase in M2/M1 microglia polarization and in the ratio of mBDNF/pro-BDNF in the hippocampus.	[76]
Oxitocin	4.2 μg/μL	Intranasally	Every other day, a total of 15 times.	Middle-aged APP/PS1 mice.	Open-field; Y-maze; novel-object recognition; water maze.	Restoration of cognitive function.	Effective clearance of Aβ from the brain into the cerebrospinal fluid; normalization of brain structure.	[110]
Ponicidin	5 or 10 mg/kg daily	No data	For 4 weeks.	5 × FAD transgenic mice (3 months old, AD model).	T-maze; Y-maze, Barnes maze; Morris water maze.	Restoration of cognitive function.	Reduced amyloid-beta (Aβ) plaque deposition and alleviated neuroinflammation and necroptosis in the brain.	[86]
Posiphen (Buntanetap)	10 or 25 mg/kg	Orally	For 1 month.	APP/PS1 mouse model of AD	Radial-arm water maze; fear conditioning; visible-platform task.	Restoration of spatial memory and improvement in contextual spatial learning.	Improvement in synaptic function.	[51]
Salicin	12.5 or 25 or 50 mg/kg	Intraperitoneally	Chronic	Mice, scopolamine-induced neurodegeneration.	Radial arm maze; novel-object recognition	Improved learning and memory.	Neuroprotective effect observed in the cortex and hippocampus.	[88]
Triazine thio-analogue (compound 4c)	0.3, 1 or 3 mg/kg	Intraperitoneally	A single administration, 60 min before the test.	Wistar rats	Novel-object recognition.	Reversal of the MK-801-induced memory deficit.	Protection against glutamate- and Aβ-induced cytotoxicity (HT-22 cells).	[91]
Tenuigeine	2, 4 or 8 mg/kg	Intragastric	28 days	Rats, streptozotocin-induced neurodegeneration.	Morris water maze.	Improvement in cognitive functions.	A reduction in the concentration of oxidative stress markers and the inhibition of changes associated with tau hyperphosphorylation.	[63]

Aβ—amyloid-β; APP—Amyloid precursor protein; BACE1—β-secretase 1; BDNF—Brain-derived neurotrophic factor; ICV—lateral ventricle injection; LPS—Lipopolysaccharide; mBDNF—mature BDNF; NLRP3—nucleotide-binding domain and leucine-rich repeat protein 3; proBDNF—precursor BDNF; VEGFC—vascular endothelial growth factor C.

**Table 3:** Potential drugs for the treatment of Alzheimer’s disease—additional data.

Drugs	Main Indication	Studies in Other Indications	Regulatory Status	Key Clinical Evidence	Magnitude of Clinical Effect	Key Limitations of Use	Ref.
Semorinemab	A potential therapy for modifying the course of AD, targeting tau pathology.	n/d	An experimental medicine, not authorized for treatment.	No significant clinical benefit.	0	No significant clinical efficacy.	[25,26,27]
Zagotenemab	A potential therapy for early symptomatic AD.	n/d	An experimental medicine, not authorized for treatment.	No slowing of clinical progression.	0	No significant clinical efficacy.	[29,30]
Tilavonemab	A potential therapy for early AD.	Progressive Supranuclear Palsy [125]	An experimental medicine, not authorized for treatment; no further research will be carried out.	No significant clinical benefit.	0	No significant clinical efficacy.	[28]
Fosgonimeton	A potential therapy for mild-to- moderate AD.	Dementia in Parkinson’s disease [126] Dementia with Lewy Dobies [127]	An experimental medicine, not authorized for treatment.	Failure to reach the endpoint.	0	No significant clinical efficacy.	[40,41]
Blarcamesine	Early AD	Rett syndrome [128] Parkinson’s Disease [126] Fragile X syndrome (FXS) [129]	An experimental medicine, not authorized for treatment. The application for authorization in the EU was withdrawn in 2026.	Insufficient data for the authorization of a medicinal product.	0	Restriction of the target group (patients without a mutation in the SIGMAR1 gene). No significant clinical efficacy. Side effects	[44,130]
Bezisterim (NE3107)	Mild-to-moderate probable AD.	Long COVID (Post-COVID-19 syndrome) Parkinson’s Disease Inflammation and insulin resistance [131]	An experimental medicine, not authorized for treatment.	No significant clinical benefit.	0	There is no data on the long-term safety of the treatment. Potential interactions with other medicines.	[49]
Posiphen (Buntanetab)	Early AD.	Parkinson’s Disease Dementia—PDD [132] Parkinson’s Disease [53]	An experimental medicine, not authorized for treatment.	Failure to reach the endpoint.	0	Only the early stage of the disease and confirmed amyloid or tau pathology. No significant clinical efficacy.	[53,54]
Triazinetrione derivative (ACD856)	AD	Traumatic Brain Injury Sleep disorders and sleep apnea Obesity Parkinson’s Disease Depression [133]	An experimental medicine, not authorized for treatment.	It is safe, has a stable pharmacokinetic profile, with rapid absorption and dose-dependent exposure. It crosses the blood–brain barrier, reaches an adequate concentration in the central nervous system and induces dose-dependent, treatment-related changes in qEEG parameters, indicating an effect on the central target.	n/d	Too early a stage of research, not enough data.	[58,134]
β-Asarone + tenuigenin	supportive therapy for AD.	n/d	An experimental medicine, not authorized for treatment.	Increases the effectiveness of memantine. Improvement in cognitive function.	0	β-Asarone raises toxicological concerns. Small study, a short follow-up period. Low bioavailability.	[68,69]

## Data Availability

The data presented in this study are available in references number [1,2,3,4,5,6,7,8,9,10,11,12,13,14,15,16,17,18,19,20,21,22,23,24,25,26,27,28,29,30,31,32,33,34,35,36,37,38,39,40,41,42,43,44,45,46,47,48,49,50,51,52,53,54,55,56,57,58,59,60,61,62,63,64,65,66,67,68,69,70,71,72,73,74,75,76,77,78,79,80,81,82,83,84,85,86,87,88,89,90,91,92,93,94,95,96,97,98,99,100,101,102,103,104,105,106,107,108,109,110,111,112,113,114,115,116,117,118,119,120,121,122,123,124,125,126,127,128,129,130,131,132,133,134,135,136,137,138]. These data were derived from the following resources available in the public domain: PubMed (https://pubmed.ncbi.nlm.nih.gov, URL accessed between July and October 2026) and Clinical trials (https://clinicaltrials.gov, URL accessed between July and October 2026).

## Abbreviations

5-HT	Serotonin
Aβ	Amyloid-β
AChE	Acetylcholinesterase
AD	Alzheimer’s disease
ADAS-Cog	Alzheimer’s Disease Assessment Scale-Cognitive
ADCS-ADL	Alzheimer’s Disease Cooperative Study—Activities of Daily Living scale
ADCS-MCI-ADL-24	24-item Alzheimer’s Disease Cooperative Study/Activities of Daily Living Scale Adapted for Patients With Mild Cognitive Impairment
ADL	Activities of Daily Living
APP	Amyloid precursor protein
ARIA-E	Amyloid-related imaging abnormalities with edema or effusions
ARIA-H	Amyloid-related imaging abnormalities with hemosiderin deposits
AQP4	Aquaporin-4
BACE1	β-secretase 1
BDNF	Brain-derived neurotrophic factor
CCR7	Chemokine receptor type 7
CDR	Clinical Dementia Rating Scale;
CDR-SB	Clinical Dementia Rating Scale–Sum of Boxes
CSF	Cerebrospinal fluid
DCE-MRI	Contrast-enhanced MRI
DPPH	2,2-diphenyl-1-picrylhydrazyl
EF	Epimedium brevicornu flavonoids
ERK	Extracellular signal-regulated kinases
ERP P300	Event-related potential P300
GST	Global Statistical Test of Cog12
IADRS	Integrated AD Rating Scale
ICV	Lateral ventricle injection
ISF	Interstitial fluid
JAK 1	Janus kinase 1
LPS	Lipopolysaccharide
MAP2K1	Mitogen-activated protein kinase 1
MAPT	Microtubule-Associated Protein Tau
mBDNF	Mature BDNF
MLKL	Mixed lineage kinase domain-like protein
MMSE	Mini-Mental State Examination
mTOR	Mechanistic target of rapamycin
NGF	Nerve growth factor
NLRP3	Nucleotide-binding domain and leucine-rich repeat protein 3
NPI	Neuropsychiatric inventory
PD-1	Programmed cell death protein
PD-L1	Programmed death-ligand 1
PPARα	Ligand of peroxisome proliferator receptor α
proBDNF	Precursor BDNF
PSEN1	Presenilin 1
PSEN2	Presenilin 2
RIPK1	Receptor-interacting protein kinase 1
STAT 1	Signal transducer and activator of transcription 1
TLR4	Toll-like receptor 4
TMT	Trail making test
TNF-α	Tumor necrosis factor α
TREM2	Triggering receptor expressed on myeloid cells 2
TRK	Receptor tyrosine kinases
VEGFC	Vascular endothelial growth factor C
WAIS	Wechsler Adult Intelligence Scale
